# Assessors’ Perspectives on the Failure to Fail Underperforming Nursing Students: A Narrative Review

**DOI:** 10.1155/nrp/6779721

**Published:** 2026-11-13

**Authors:** Adele Taylor, Kevin Hambridge

**Affiliations:** ^1^ Royal Devon University Healthcare NHS Foundation Trust Barrack Road Exeter EX2 5DW UK royaldevon.nhs.uk; ^2^ The Exeter School of Nursing InFocus Charity University of Plymouth Topsham Road, Countess Weir Exeter Devon EX2 6HA UK plymouth.ac.uk; ^3^ University of Plymouth Plymouth PL4 8AA Devon UK plymouth.ac.uk

**Keywords:** assess, competence, failure to fail, narrative review, student nurse, underperform

## Abstract

**Aims:**

The aim of this narrative review is to explore reasons for the failure to fail of underperforming, nursing students, from the assessor perspective.

**Background:**

When assessing student nurses on practice placements, assessors often have difficulty assigning a failing grade to students who fail to demonstrate competence in accordance with practice standards. Failure to fail is the act of passing nursing students on their clinical placement who do not display acceptable clinical performance. This issue can have considerable consequences for patient and staff safety, as well as legal implications for the workplace and the nursing profession.

**Design:**

A mixed‐methods narrative review utilises thematic synthesis, following Thomas and Harden’s (2008) three‐stage approach of coding, development of descriptive themes and generation of analytical themes.

**Methods:**

A search of CINAHL, Emcare, Ovid and ProQuest—British Nursing Index and Google Scholar was conducted, and hand‐searching techniques were utilised. All data were sourced between November 2023 and January 2024. English‐language literature published since 2019, focusing on the assessment of underperforming students from the assessor’s perspective, was reviewed.

**Results:**

Eleven papers were included in the review, nine of which were qualitative and two quantitative papers. Thematic synthesis was used to identify four main themes: educational assessment and feedback, student assessment, practical constraints and educational psychology.

**Conclusion:**

Failure to fail is an ongoing issue and was evidenced in the literature from a variety of high‐income countries. Assessors experienced various challenges in failing underperforming students. It is essential for assessors to have the support of practice educators and university faculties, to have protected time to complete assessments and associated documentation and to have access to further education and training.

## 1. Introduction

The purpose of pre‐registration nursing education is to prepare student nurses for future practice as registrants (Nursing and Midwifery Council [NMC] [1]). Clinical placements are a significant element of student nurse education (NMC [2]; Nyoni et al. [3]) for students to demonstrate professional values, knowledge, skills and attitude. This allows them to progress to the next stage of training and ultimately to become registered nurses (NMC [4]). The UK student nurse pre‐registration programme takes 3–4 years to complete, including 2300 practice hours (NMC [1]). Increasing the number of nurses, while also ensuring they have the correct skills, knowledge and attitudes is crucial to producing proficient nurses capable of providing safe and high‐quality care to patients (World Health Organisation [WHO] [5]).

In the UK, practice supervisors and assessors support and assess students in clinical placements (NMC [6]). Students’ assessments vary internationally, as do the titles and roles of those who assess students. There is consistency in that registered nurses supervise, support and assess students on clinical placements with some triangulation and input from clinical colleagues and university academics (Immonen et al. [7]). To ensure clarity, all personnel who evaluate students in practice are referred to as ‘assessors’ in this review.

The assessment of student nurses must be robust and evidence‐based, encompassing integrity (NMC [2]). As part of their professional code, nurses are expected to recognise and report unsafe care delivered by others, including students (Tanicala et al. [8]; NMC [9]). Assessors are crucial in identifying and managing underperforming students, and the possibility of students becoming registered nurses who lack the necessary clinical skills and attitude is of significant concern (Yepes‐Rios et al. [10]). It is vital that assessors receive adequate additional support, both from practice and partner universities to monitor student progress, ensure assessment meetings are effective and timely, and be capable of having difficult conversations with failing students when necessary (Maxwell et al. [11]).

Failure to fail is a term describing the practice of avoiding having to fail a student who does not demonstrate satisfactory clinical performance (Hughes et al. [12]). Hughes et al. [13] reported that although 73.8% of assessors did not generally believe in giving students the benefit of the doubt, 23.5% of the participating assessors had done so, either sometimes or often. Passing tolerance decreased with the level of experience, with 12% passing underperforming first‐year students, 4.7% passing underperforming second‐year students and 1.3% passing underperforming final‐year students. Nearly half of participants recruited to Adkins and Aucoin [14] quantitative study answered positively to questions that indicated a history of failure to fail. Natterøy et al. [15] reported that few participants had formally escalated concerns, or failed students who were not achieving, but two participants stated that in retrospect, they should have failed underperforming students.

Failure to fail has been recognised within nursing education for over 2 decades and can have significant consequences for the student, clinical colleagues, patients, relatives, the healthcare trust and the NMC (Scholes and Albarran [16]). The reasons for failure to fail must therefore be understood by those involved in student nurse assessment to fully support and enable assessors to effectively fulfil their roles.

## 2. Methods

### 2.1. Aim

The aim of this narrative review is to explore reasons for the failure to fail of underperforming, nursing students, from the assessor perspective.

### 2.2. Design

The design of this review is a mixed‐methods narrative review utilising a thematic analysis. A narrative review methodology was considered most appropriate for addressing the review aim, as it facilitated the synthesis of evidence from a diverse body of literature exploring factors influencing decisions to fail underperforming student nurses. Although systematic review methods informed the search, screening and appraisal processes, the review was primarily undertaken by a single reviewer, with supervisory oversight of selected decisions. Therefore, the review is presented as a narrative review rather than a systematic review, reflecting the pragmatic approach taken while maintaining methodological transparency.

### 2.3. Search Strategy

The PEO framework (Bettany‐Saltikov [17]) was utilised for the purposes of this review to design the following research question:

‘To explore the barriers and enablers (E) of failing (O) underperforming student nurses (P)’.

While specific terms relating to barriers and enablers were not used, the exposure component was captured through search terms relating to assessment roles and assessment processes (e.g., assessor^∗^, mentor^∗^ and practice assessor), enabling identification of studies exploring factors that influence decisions to fail underperforming students.

#### 2.3.1. Search Terms

The search method was discussed with information specialists ensuring that all relevant studies were included, and that databases and search terms were used effectively (Lefebvre [18]). The search terms used were as follows: (‘student nurses’ OR ‘nursing students’ OR ‘student nurse’ OR ‘nursing student’ OR ‘undergraduate nurse’ OR ‘pre‐registration’) AND (‘failure to fail’ OR fail^∗^ OR underperform^∗^ OR competenc^∗^ OR mentor^∗^ OR NMC OR ‘Nursing and Midwifery Council’ OR assess^∗^ OR ‘practice assessor’ OR ‘practice supervisor’). Subject headings and keywords were combined using Boolean operators AND/OR to allow for a more targeted search (EBSCO Connect [19]).

#### 2.3.2. Databases Searched

A search was conducted using the following databases: CINAHL, Emcare, Ovid and ProQuest—British Nursing Index. Using a combination of the databases aimed to enhance the comprehensiveness of the review (Bramer et al. [20]). An academic search engine was also used, which identified some grey literature. Citation chasing was carried out by reviewing the bibliography of references for each work (backward citation chasing) and searching Google Scholar (forward citation chasing) (Cooper et al. [21]). The search was performed between November 2023 and January 2024.

### 2.4. Inclusion and Exclusion Criteria

The inclusion and exclusion criteria can be seen in Table 1.

**TABLE 1:** Inclusion and exclusion criteria.

Included	Excluded	Rationale for choice
Studies including student nurse, nursing student, undergraduate nurse	Studies concerning other types of students and not related to nursing.	The focus of the review being failure to fail in student nurses. The author did not wish to include literature concerning other healthcare students. Various key words were included in the search to capture different terminology used to describe ‘student nurses’ and also capture the concept of assessment of those students, failure and underperformance.

Studies from the assessor perspective	Studies from the student perspective	The aim of the review was to explore the assessor experience of the barriers and enablers of failing underperforming student nurses; therefore, literature from the student’s perspective was excluded.

Studies within the date range of 2019–2024	Studies published outside the specified date range, or those within it that reported data collection prior to 2018, were excluded.	The author wished to review evidence from the last 5 years (at the time of writing) and not overlap with any previous systematic reviews conducted.

Studies written in English	Studies in other languages	The author did not have the ability, time or resources to translate literature.

Studies included from UK, Europe, Australia, New Zealand, USA and Canada.	Studies from the rest of the world	Literature was included from ‘high‐income countries’ due to similarities in healthcare systems and student nurse education.

All primary research, including qualitative, quantitative and mixed methods.	Secondary resources	Primary research papers subjected to a thorough process of peer review, in order to review evidence of a greater level of quality and dependability than secondary sources.

### 2.5. Study Selection

The study selection and critical appraisal were conducted primarily by the author. To enhance methodological rigour and reduce potential bias, a sample of papers was independently reviewed by the author’s supervisor, and decisions were discussed throughout the review process. The study selection process is summarised in the PRISMA 2020 flow diagram (Figure 1).

**FIGURE 1:**
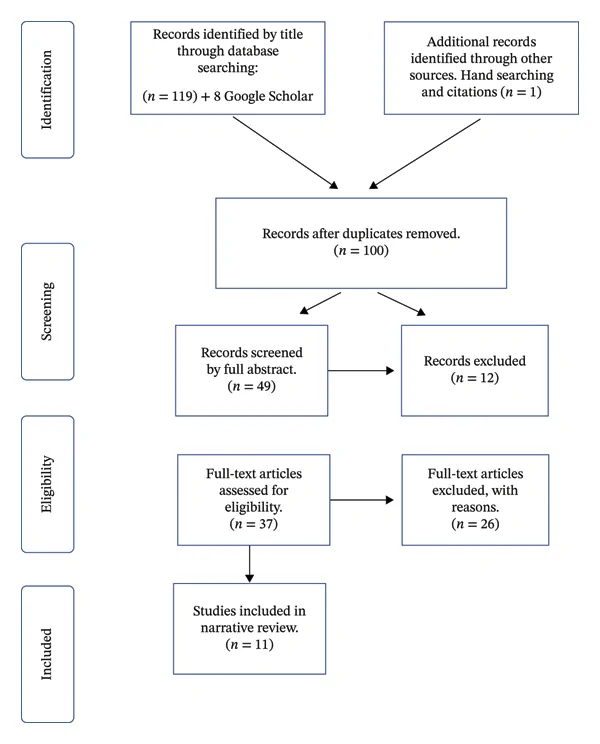
PRISMA 2020 flow diagram of study selection (Page et al. [22]).

Following the search of databases, 127 papers were selected by title. The removal of duplicates reduced the reference list to 100 titles. Of the 100 papers, 63 were eliminated following abstract screening, 37 articles underwent a full‐text evaluation, and 26 were rejected based on relevance to the topic and inclusion/exclusion criteria. The remaining 11 articles were appraised using the appropriate appraisal tools. Following appraisal, no studies were excluded, as the review aimed to provide a comprehensive synthesis of the available evidence. The quality of individual studies was considered when interpreting findings and drawing conclusions.

A table of the included papers can be found in Table 2.

**TABLE 2:** Table of included papers in review.

Reference	Country	Aims	Methods	Sample size	Setting	Findings	Analysis techniques
Aase et al. [23] Exploring the formal assessment discussions in clinical nursing education: An observational study.	Norway	To explore the characteristics of the formal assessment discussions taking place during first‐year students’ clinical education in nursing homes.	Exploratory qualitative study. Passive observations of 24 assessment discussions with nursing students, mentors and educators.	*n* = 17	Three separate nursing homes in a single Norwegian municipality. Data collected in February and March 2019	Three themes identified regarding characteristics of the formal assessment discussions: • Adverse variability in structuring, weighting of theoretical content and pedagogical approach; • Limited three‐part dialogue constrains feedback and reflection • Restricted grounds for assessment leave the nurse educators with a dominant role.	Thematic analysis

Adkins and Aucoin [14] Failure to fail—Factors affecting faculty decisions to pass underperforming nursing students in the clinical setting: A quantitative study	USA	To explore the relationship between face‐implicating factors and faculty’s likelihood of failing students in the clinical setting who do not meet passing criteria.	Quantitative descriptive design online survey.	*n* = 327	Snowball response of 327 pre‐registration clinical nursing faculty from associate and baccalaureate nursing programmes. Participants were clinical instructors with at least 3 years’ experience. Data collected between 14 April and 16 May 2020.	Respondents who did not commit failure to fail were less affected by the face‐implicating factors than those who committed failure to fail.	Statistical analysis

Borren et al. [24] A qualitative analysis investigating competence assessment of undergraduate nursing students	New Zealand	To identify current competence assessment practice and determine how competence assessment is constructed in order to reflect student development.	Qualitative exploratory descriptive design. 10 semistructured interviews conducted with nurse educators. No date specified for data collection.	*n* = 10	Participants had direct involvement with student assessment process over nine tertiary institutions in NZ.	Three themes reported: • Clinical assessment pedagogy • Measure of competence • Relational assessment practice.	Thematic analysis

Christiansen et al. [25] Challenges in the assessment of nursing students in clinical placements: Exploring perceptions among nurse mentors	Norway	To explore how nurse mentors experience the assessment of nursing students in clinical placements	The study is qualitative with an explorative and descriptive design exploring how nurse mentors experience the assessment of nursing students in clinical placements at hospitals and in municipal health care. 19 individual qualitative interviews and four focus group interviews conducted.	*n* = 33	33 nurse mentors from various levels and fields of nursing education recruited to participate from one Norwegian university. Data collected autumn 2017 and spring 2018.	• Feedback was an integrated part of the formative assessment. • Divergent views on student’s competence which challenged the credibility of student assessment. • Perceptions of nursing values as a criterion and the impact on assessment.	Thematic analysis

Devlin and Duggan [26] An evaluation of nurses′ experiences of mentoring pre‐registration students	UK	• Gain an understanding of registered nurse mentors’ experiences in practice when mentoring pre‐registration nursing students • Evaluate how mentors perceived the support they were given • Highlight to all relevant stakeholders, including AEIs and practice education teams, how mentors need to adapt in practice while supporting and assessing students	Qualitative research comprising 6 semistructured interviews across 1 UK healthcare trust.	*n* = 6	6 participants from one NHS Trust. Data taken after NMC SSSA implemented from 2018.	• Engagement (barriers versus strategies) • Support (inclusivity versus exclusivity) • Lack of recognition	Thematic analysis

Hughes et al. [13] Just how bad does it have to be? Industry and academic assessors′ experiences of failing to fail—A descriptive study	Australia	To describe assessors′ experiences of grading nursing student performances in clinical courses when that performance was not a clear pass or fail.	Quantitative online survey with 149 participants.	*N* = 149	Survey distributed via email to educators in all states and territories in Australia. Data collected in March 2018	• Evidence of failure to fail • Coercive student behaviours • Impact of placement culture • Inadequate time to assess students	Statistical analysis

Mathisen et al. [27] Practice education facilitators perceptions and experiences of their role in the clinical learning environment for nursing students: a qualitative study	Norway	To explore how practice education facilitators can contribute to strengthen the clinical learning environments for nursing students.	Qualitative explorative design, with in‐depth interviews conducted with practice education facilitators from three different universities.	*n* = 12	12 participants from three different universities located in southeast, mid‐ and northern Norway. Data collected spring 2021.	Four themes: • Coherence between theory and practice • Student support and guidance during placement • Supporting the supervisors to support the students • Factors influencing the practice education facilitators’ performance in their role.	Thematic analysis

Natterøy et al. [15] Nurse mentors′ experiences with suitability assessments in clinical placement: A qualitative study	Norway	To explore mentors’ experiences with assessing the suitability of nursing students in clinical placements	Qualitative exploratory design. Individual, semistructured interviews conducted with 17 student mentors.	*n* = 17	Participants from 3 different hospitals in Norway. Data collected in May and June 2022	Four themes: • The understanding of suitability • Experiencing borderline performance • The heavy responsibility of doing the assessment • The importance of support	Thematic analysis

Rees et al. [28] Power and resistance in feedback during work‐integrated learning: contesting traditional student‐supervisor asymmetries, Assessment & Evaluation in Higher Education	Australia	To answer how do students and supervisors narrate student power and resistance within work‐integrated learning feedback narratives?	Qualitative feedback narratives collected from education students and supervisors from six disciplines, including 3 nursing students and 4 nursing supervisors.	*n* = 94	One Australian university. No date specified for data collection.	• Student enactment of power in the feedback relationship. • Supervisors’ power‐sharing with students in the feedback relationship • Students’ resistance to supervisor power in the feedback relationship	Thematic and narrative analysis

Rodger and Juckes [29] Managing at risk nursing students: The clinical instructor experience	Canada	To explore the experience of the clinical instructor when managing students at risk for failure.	Qualitative focus groups with 7 clinical instructors. No date specified for data collection.	*n* = 7	Seven instructors recruited across two sites of a Canadian nursing college.	• Supporting student learning while ensuring patient safety. • Balancing responsibilities and professional obligations. • Feeling physically and emotionally drained. • Experiencing self‐doubt in making decisions. • Seeking and finding support.	Thematic analysis

Whaley et al. [30] Preparing nurse educators for NMC standards for student supervision and assessment: the impact 4 years on.	UK	To identify the enablers and challenges that influence the new PA/PS/AA roles, with emphasis placed on educators’ ground‐level experiences	Qualitative online survey to explore the experiences of 48 practice assessors and practice supervisors since the implementation of the NMC standards 4 years previously.	*n* = 48	Participants were educators across one integrated care system (ICS) within the Midlands, UK. Data collected between October and November 2021	• Variation in levels of preparation • Outdated knowledge • Prohibitive workloads • Role conflict	Thematic analysis

### 2.6. Quality Appraisal

The qualitative appraisal tool (CASP [31]) and the Critical Appraisal of Cross‐Sectional Study appraisal tool were utilised (Centre for Evidence‐Based Management [32]). The final papers were reviewed and appraised. To mitigate risk of bias, a selection of papers was independently reviewed by the author’s supervisor. Critical appraisal indicated that the included studies were generally methodologically robust, although some methodological limitations and areas of uncertainty were identified. Full appraisal findings are presented in Tables 3 and 4.

**TABLE 3:** Results of CASP appraisals for qualitative papers.

Paper	CASP checklist for qualitative research									
	1	2	3	4	5	6	7	8	9	10
Aase et al. [23]	Y	Y	Y	Y	Y	Y	Y	Y	Y	Y
Borren et al. [24]	Y	Y	Y	Y	Y	Y	Y	Y	Y	Y
Christiansen [25]	Y	Y	Y	Y	Y	Y	Y	Y	Y	Y
Devlin and Duggan [26]	Y	Y	Y	Y	Y	Y	Y	Y	Y	Y
Mathisen et al. [27]	Y	Y	Y	Y	Y	CAN’T TELL	Y	Y	Y	Y
Natterøy et al. [15]	Y	Y	Y	Y	Y	Y	Y	Y	Y	Y
Rees et al. [28]	Y	Y	Y	Y	Y	N	Y	Y	Y	Y
Rodger and Juckes [29]	Y	Y	Y	Y	Y	Y	Y	Y	Y	Y
Whaley et al. [30]	Y	Y	Y	Y	Y	CAN’T TELL	Y	CAN’T TELL	Y	Y

**TABLE 4:** Results of critical appraisals of cross‐sectional studies.

Paper	Critical appraisal of a cross‐sectional study (survey)											
	1	2	3	4	5	6	7	8	9	10	11	12
Adkins and Aucoin [14]	Y	Y	Y	Y	Y	Y	Y	Y	Y	N	Y	Y
Hughes et al. [13]	Y	Y	Y	Y	CAN’T TELL	N	CAN’T TELL	Y	N	N	Y	Y

### 2.7. Data Synthesis

A thematic synthesis was undertaken following the approach described by Thomas and Harden [33]. Findings from the included studies were coded, with data extracts identified through colour‐coded highlighting and assigned initial codes. Similar codes were grouped into descriptive themes representing recurring patterns across studies. A third stage of synthesis involved interpreting these themes to synthesise findings across the studies and explore their meaning in relation to the review.

## 3. Results

### 3.1. Study Characteristics

From the 11 articles chosen for the review, nine were qualitative (Devlin and Duggan [26]; Rees et al. [28]; Christiansen et al. [25]; Rodger and Juckes [29]; Aase et al. [23]; Borren et al. [24]; Mathisen et al. [27]; Natterøy et al. [15]; Whaley et al. [30]) and two were quantitative surveys (Hughes et al. [13]; Adkins and Aucoin [14]). Four of the papers were from Norway (Christiansen et al. [25]; Aase et al. [23]; Mathisen et al. [27]; Natterøy et al. [15]); two from Australia (Hughes et al. [13]; Rees et al. [28]) and the UK (Devlin and Duggan [26]; Whaley et al. [30]); and one from Canada (Rodger and Juckes [29]), New Zealand (Borren et al. [24]) and the USA (Adkins and Aucoin [14]).

### 3.2. The Findings

An analysis of the literature related to failure to fail identified four themes: educational assessment and feedback, student assessment, practical constraints and educational psychology.

#### 3.2.1. Educational Assessment and Feedback

Nine papers highlighted a struggle to understand the assessment process, documentation and defining competence (Hughes et al. [13]; Devlin and Duggan [26]; Christiansen et al. [25]; Roger and Jukes [29]; Adkins and Aucoin [14]; Borren et al. [24]; Mathisen et al. [27]; Natterøy et al. [15]; Whaley et al. [30]):

‘I find the way the portfolio is written very waffly’ (Devlin and Duggan [26]).

Despite unfamiliarity with formal suitability or competence assessment criteria, most assessors understood the concepts of competence, expectations and implications. However, differing views on competence led to subjectivity and variability in assessments (Christiansen et al. [25]; Borren et al. [24]):

‘It will just be my own thoughts because I don’t quite know what the word “suitability” [competence] means’ (Natterøy et al. [15]).

Insufficient understanding of assessment processes was exacerbated by a lack of standardisation, recent changes to assessment standards in some countries and variation in assessment documentation across institutions, which further perplexed assessors supporting students from multiple universities (Borren et al. [27]; Whaley et al. [30]):

‘it requires more … paperwork and as the students also at times look for guidance from us, that makes me a bit uncertain’ (Whaley et al. [30]).

The practice educator was seen as a supportive role, facilitating the assessment process by acting as a liaison between the university and placement. This led to the early detection of students encountering difficulties and, consequently, reduced the risk of failure to fail (Devlin and Duggan [26]; Mathisen et al. [27]).

Despite educator and managerial support, some assessors felt overburdened due to too few nurses undertaking the role (Devlin and Duggan [26]), whereas the presence of a positive learning culture was considered crucial, involving all staff members in a collective responsibility for student progress, supervision and feedback (Hughes et al. [13]; Devlin and Duggan [26]).

Assessors experienced self‐doubt and decision‐making difficulties, causing emotional stress and leading them to question their support of the student. The need for greater confidence in decision‐making when issuing a failing grade to an underperforming student was identified, along with the need for enhanced assessor education (Hughes et al. [13]; Devlin and Duggan [26]; Roger and Jukes [29]; Adkins and Aucoin [14]; Borren et al. [24]; Natterøy et al. [15]; Whaley et al. [30]):

‘an annual update/meeting would be nice to avail us of any changes/updates rather than yet finding out from the students’ (Whaley et al. [30]).

Assessors were divided on their self‐assessment of competence, but many reported that updates and specific education around the assessment of borderline students improved confidence, decreased feelings of self‐doubt and reduced failure to fail (Hughes et al. [13]; Roger and Jukes [29]; Natterøy et al. [15]; Whaley et al. [30]). Those receiving support felt empowered, while others acknowledged the benefits and anticipated a reduction in failure to fail (Hughes et al. [13]). However, workload demands limited assessors’ ability to attend educational sessions (Roger and Jukes [29]).

Assessing competence or suitability was complex, as it encompasses clinical skills, knowledge, professional attitudes, communication, the capacity for compassion and empathy towards patients (Hughes et al. [13]; Christiansen et al. [25]; Natterøy et al. [15]). Psychosocial elements were considered more difficult to measure and define, but seen as important, even if they did not feature on the assessment criteria (Christiansen et al. [25]). Assessors were divided on failing a student solely due to their professional attitude (Hughes et al. [13]). Assessor intuition played a part, with some describing a sense or feeling when something was amiss, comparing it to clinical reasoning when caring for patients and adding an additional standard of quality; asking if they would like this student caring for their own loved ones (Christiansen et al. [25]; Natterøy et al. [15]). Assessors tolerated student mistakes if the student was reflective and accepting of feedback; however, unsafe student practices that compromised patient care were not tolerated (Rodger and Juckes [29]; Natterøy et al. [15]):

‘There is a point where you as a nurse say, we can′t keep doing this, this isn’t okay, no matter how hard you [the student] is trying, you are not meeting the expectations’ (Rodger and Juckes [29]).

The importance of providing early support to assessors in managing underperforming students was recognised, as a lack of timely assistance, particularly in the absence of a second opinion prior to the midpoint assessment, contributed to reluctance or failure to fail (Mathisen et al. [27]). The appeals process was perceived as biased in favour of students, with reports of successful student appeals resulting in assessor decisions being overturned (Hughes et al. [13]).

Four studies highlighted the importance of formative assessments and dialogue between students and assessors (Hughes et al. [13]; Christiansen et al. [25]; Aase et al. [23]; Borren et al. [24]). Time constraints and limited communication diminished both the quantity of feedback and the quality of learning during formal assessments in clinical placements (Hughes et al. [13]; Christiansen et al. [25]; Aase et al. [23]):

‘I feel we don’t have time to talk to the students every day’ (Christiansen et al. [25]).

#### 3.2.2. Student Assessment

While the Educational Assessment and Feedback theme centred on assessment processes, assessor competence and feedback practices, this theme highlighted the interpersonal, collaborative and organisational factors that influenced assessment decision‐making.

The interactions and relationships between students and their assessors play a crucial role in practice‐based learning (Davis et al. [34]; King et al. [35]). Collaboration and support between colleagues, nurse educators and university faculties were identified as important aspects, ‘another pair of eyes’ to ensure an unbiased and valid assessment, particularly for borderline students (Devlin and Duggan [26]; Christiansen et al. [25]; Rodger and Juckes [29]; Borren et al. [24]; Mathisen et al. [27]; Natterøy et al. [15]). In the absence of colleague support, assessors reported feeling isolated in carrying the weight of assessment responsibilities and expressed a need for greater collaboration with university lecturers (Christiansen et al. [25]).

‘Just being able to share my thought processes and judgments of the students, to have somebody with some experience validate what I am saying, or what I am seeing or guide me’ (Rodger and Juckes [29]).

While some assessors lacked awareness of the academic assessor or broader university role (Devlin and Duggan [26]; Whaley et al. [30]), many valued the support provided by the university, felt confident initiating contact and relied on it to inform their decision‐making (Hughes et al. [13]; Rodger and Juckes [29]; Natterøy et al. [15]). The value of the collaboration was dependant on individual tutors (Natterøy et al. [15]), with some reports of lack of support resulting in personal or psychological distress and feelings of isolation (Devlin and Duggan [26]; Christiansen et al. [25]). Assessors expressed a need for stronger connections between practice colleagues and educational institutions, along with greater university visibility and involvement, to ensure adequate support (Whaley et al. [30]). Assessors valued the role of the practice educator, favouring familiar and readily accessible support over that provided by geographically dispersed academic tutors (Mathisen et al. [27]).

Power differential between students and assessors was acknowledged, highlighting the potential influence this imbalance can have on assessment dynamics and decision‐making (Rees et al. [28]; Borren et al. [24]; Natterøy et al. [15]). Assessors recognised their power and influence in terms of student progression, with some expressing concern about wielding such power (Borren et al. [24]; Natterøy et al. [15]). Although students were often perceived as powerless, some were observed to resist assessor authority by disregarding feedback, expressing disagreement, reacting inappropriately or openly challenging assessments (Rees et al. [28]; Natterøy et al. [15]):

‘she sort of just sat there and rolled her eyes…I don’t trust your clinical judgement … I want somebody else to do it’ (Rees et al. [28]).

Some students also exhibited challenging, manipulative or abusive behaviour. These behaviours intensified when students disclosed life circumstances such as visa requirements, care duties, prior nursing experience or financial concerns (Hughes et al. [13]; Rees et al. [28]; Rodger and Juckes [29]). Assessors felt concern for students and were interested in establishing the factors affecting their behaviour (Natterøy et al. [15]). A lack of insight was identified as a contributing factor in some students’ inability or reluctance to take responsibility, with blame occasionally redirected towards assessors through complaints or, in extreme cases, acts of aggression (Hughes et al. [13]; Rees et al. [28]; Rodger and Juckes [29]).

#### 3.2.3. Practical Constraints

Workload pressures were a theme in six of the studied papers (Hughes et al. [13]; Devlin and Duggan [26]; Christiansen et al. [25]; Aase et al. [23]; Borren et al. [24]; Whaley et al. [30]). While time constraints influenced feedback, learning opportunities and assessment processes, they were also identified as a broader organisational issue linked to workload pressures, competing clinical priorities and administrative demands. Participants described being overwhelmed in their work, with lack of time repeatedly cited as a barrier to effective supervision and assessment (Hughes et al. [13]; Christiansen et al. [25]; Whaley et al. [30]). Completing the subsequent documentation was seen as extra work, difficult to manage in addition to patient care and other registered nurse responsibilities, and was often completed at home during personal time (Devlin and Duggan [26]; Whaley et al. [30]):

‘Short staffing…high patient acuity…meeting the needs of the patients and the organisation, has proven difficult to enable sufficient time to discuss, teach and complete student documents and interviews’ (Whaley et al. [30]).

Assessors raised concerns about the validity of assessments, as they felt they had not spent sufficient time directly observing their students (Aase et al. [23]; Borren et al. [24]):

‘Time constraints on the ward have become worse over the years and at times I feel that the students get used as a pair of hands rather than a learner’ (Whaley et al. [30]).

UK‐based assessors reported feeling undervalued, with some expressing dismay that their previously earned mentoring qualifications had become obsolete under the NMC [2] SSSA (Devlin and Duggan [26]; Whaley et al. [30]). The perceived lack of recognition from management, coupled with expectations to undertake additional responsibilities without remuneration, contributed to feelings of resentment (Devlin and Duggan [26]; Whaley et al. [30]). Although some assessors acknowledged these duties as inherent to their role as registered nurses, many considered them unjust, reinforcing negative perceptions of the assessor role:

‘you don’t get paid any more for doing it but you are expected to do it’ (Devlin and Duggan [26]).

#### 3.2.4. Educational Psychology

The findings highlighted several psychological and emotional factors that influenced assessors’ decision‐making when evaluating underperforming students. For some assessors, supporting nursing students and observing their development evoked a tangible feeling of pride, reward and achievement (Hughes et al. [13]; Whaley et al. [30]). However, many reported role conflict between workload pressures and effective learner supervision and assessment (Hughes et al. [13]; Devlin and Duggan [26]; Aase et al. [23]; Borren et al. [24]; Mathisen et al. [27]; Whaley et al. [30]). The emotional cost of assessment was also noted, with assessors reporting emotional distress for unsuccessful students, guilt over their investment and the weight of potentially ending a student’s career (Rodger and Juckes [29]; Adkins and Aucoin [14]; Borren et al. [24]; Mathisen et al. [27]; Natterøy et al. [15]). Developing personal connections with underperforming students was perceived as particularly challenging, given assessors’ awareness of students’ individual, familial and financial circumstances (Hughes et al. [13]; Rodger and Juckes [29]).

Adkins and Aucoin [14] suggested that these emotional responses may contribute to failure‐to‐fail behaviours through face‐implicating factors, whereby assessors can be reluctant to deliver negative judgements because of concerns about their own self‐image, professional reputation, relationships with students or the emotional impact of failing them. They reported that many themes for failure to fail were synonymous with existing literature on face‐implicating factors, commonly known as the ‘MUM effect’, whereby individuals avoid delivering unwelcome messages because of concerns about damaging relationships, causing distress or being viewed negatively (Dibble [36]; Dibble and Sharkey [37]). The similarities between Adkins and Aucoin’s [14] findings and the ‘MUM effect’ literature are outlined in Table 5.

**TABLE 5:** Identified themes from Adkins and Aucoin [14] and their comparison to the MUM effect in the literature.

Failure to fail themes	Face‐related concerns and the MUM effect in the literature
Concern about student reaction	Fear of an emotional reaction from the receiver Fear of causing distress to the receiver

Fear of being negatively evaluated	Fear of a negative evaluation

Emotional and moral toll	Desire to prevent a negative mood

Lack of confidence	Fear of the expectation to know more than one does

Role conflict or confusion	Fear of voicing one’s own emotions

Assessors who had not previously engaged in failure to fail expressed stronger disagreement with face‐related concerns; reported being unaffected by pressures from colleagues, universities or students’ reactions; demonstrated greater confidence in their decisions; and did not interpret student failure as a reflection of their own competence or compassion (Rodger and Juckes [29]; Adkins and Aucoin [14]; Mathisen et al. [27]).

## 4. Discussion

### 4.1. Interpretation of Findings

This review explored the factors that facilitate or hinder the failure of underperforming student nurses. Following comprehensive literature searches, the author justified the inclusion of studies from high‐income countries comparable to the UK, due to a lack of recent UK‐based publications. The findings suggest that assessor education and competence, student assessment practices, practical constraints and principles of educational psychology all influence the accuracy of student assessment. Consistent with the review findings, several themes identified in contemporary studies were also evident in earlier literature, suggesting that barriers associated with failing underperforming student nurses have remained persistent over time.

Three papers (Hughes et al. [13]; Adkins and Aucoin [14]; Natterøy et al. [15]) reported that some assessors admitted to passing incompetent students or giving them the benefit of the doubt‐findings consistent with prior research (Watson and Harris [38]; Duffy [39]; Gainsbury [40]; Hunt et al. [41]). This issue has persisted in British literature over the last 2 decades and is neither unique to the UK or the nursing profession (Scanlan et al. [42]; Hughes et al. [13]; Adkins and Aucoin [14]; Fisher et al. [43]; Natterøy et al. [15]; Scarff et al. [44]). Irrespective of country, assessment framework or discipline, these reports have sustained concerns that some students who qualify are not fit to enter the profession (Natterøy et al. [15]). This can lead to costly, extended, supernumerary periods upon qualification, unsafe practice, increased clinical errors and subsequent removal from the nursing register.

Assessors faced significant barriers in evaluating students in relation to assessment criteria, terminology and expectations (Hughes et al. [13]; Devlin and Duggan [26]; Christiansen et al. [25]; Roger and Jukes [29]; Adkins and Aucoin [14]; Borren et al. [24]; Mathisen et al. [27]; Natterøy et al. [15]; Whaley et al. [30]). This was exacerbated by a lack of standardisation and national assessment tools and recent changes to assessment standards in some countries (Borren et al. [24]; Whaley et al. [30]).

To ensure robust and equitable evaluation, assessors are expected to demonstrate a strong understanding of required proficiencies and course outcomes (NMC [45]; NMC [6]). Practice‐based assessments should be transparent, rigorous and fair, serving two key purposes: formative assessment for ongoing feedback and learning, and summative assessment to determine overall competence (Price [46]).

In the UK, all practice assessors must complete some level of preparation to undertake the role, but the level and content of the training are at the discretion of the Approved Education Institutions (AEIs) and their practice partners, leading to variability and a lack of consistency (NMC [47]). Assessors are expected to maintain up‐to‐date knowledge and skills, and the NMC [47] indicates that reflection on the assessor role as part of the revalidation process may be sufficient, as there is no formal requirement for mandatory updates.

This review highlights a clear need for enhanced training and support for assessors, citing persistent gaps in understanding the assessment process, navigating documentation and defining competence (Hughes et al. [13]; Devlin and Duggan [26]; Christiansen et al. [25]; Roger and Jukes [29]; Adkins and Aucoin [14]; Borren et al. [24]; Mathisen et al. [27]; Natterøy et al. [15]; Whaley et al. [30]). However, access to such support is often hindered by systemic pressures, including seasonal demand, reduced funding and workforce shortages (Merrifield [48]; HEE [49]; Devereux [50]).

The complexities of defining and assessing competence have been widely explored in the literature (Takase and Teraoka [51]; Fukada [52]; Christensen et al. [25]; Munangatire and McInerney [53]). Nursing competency as outlined by Takase and Teraoka [51] encompasses three theories: behaviourism (demonstrating core skills), trait theory (attributes such as knowledge and critical thinking) and holism (a collection of elements, including knowledge, skills and values). The psychosocial elements, such as attitudes and behaviours, can be more challenging to assess, influenced by factors such as stress, coping strategies and motivation (Morrell‐Scott [54]). Their subjective and interpretive nature makes them difficult to quantify with the same precision as clinical skills or knowledge (Christiansen et al. [25]; Natterøy et al. [15]). Duffy [55] highlights that competence issues often span cognitive, emotional and psychomotor domains, and that many failing students lack the insight necessary to accept feedback and implement change—frustrating mentors and limiting the effectiveness of supportive interventions. Given that reflection, critical thinking, self‐awareness and professional development are essential in nursing (NMC [56]), insight is crucial for identifying improvement areas, adapting to new practices and delivering high‐quality patient care.

The review highlighted assessors’ lack of time to complete assessments effectively (Hughes et al. [13]; Christiansen et al. [25]; Aase et al. [23]), a finding consistent with historical research (Watson and Harris [38]).

In addition to excessive workloads and time constraints, the additional burden of assessing students, particularly associated documentation, was perceived as challenging to manage (Devlin and Duggan [26]; Christiansen et al. [25]; Aase et al. [23]; Borren et al. [24]; Natterøy et al. [15]; Whaley et al. [30]). Supporting underperforming students further intensified this burden, leading to an overwhelming, increased workload for assessors due to additional meetings, feedback sessions, action planning and extensive assessment paperwork‐often completed without any extra time allocated (Duffy [39]; Scholes and Albarran [16]; Duffy [55]; Whaley et al. [30]). Ongoing nursing shortages further exacerbate these pressures by increasing clinical workload and reducing the time available for supervision and assessment. One strategy to address nursing workforce shortages and increase the future supply of registered nurses is to increase the recruitment of student nurses (NHS England [57]), as well as increase clinical placement capacity to accommodate them. However, this can inadvertently increase the supervisory load and dilute the quality of supervision and assessment (Williamson et al. [58]).

Failure to recognise the increased workload and emotional demands associated with the assessor role can exacerbate assessor anxiety (Pettit and Stephen [59]). This is particularly concerning given NHS staff surveys have highlighted high levels of work‐related stress and burnout (NHS England [60]). To mitigate this emotional burden and promote wellbeing, assessors require access to adequate training, structured support and restorative clinical supervision.

Workload pressures continue to undermine the quality of student learning, supervision and assessment (Grimwood [61]). The Safe Learning Environment Charter (SLEC) (NHS England [62]), developed in alignment with the NHS People Promise [63], outlines key recommendations to promote supportive learning environments that enable learners to become competent, skilled and safe practitioners. Among these is the need for assessors to be allocated protected time for completing assessments, maintaining documentation and attending training on assessment standards, documentation practices and performance monitoring (NHS England [62]). There is also a clear need to safeguard and expand the role of practice educators, who may offer a cost‐effective solution by reducing supernumerary time, mitigating the consequences of poor practice and strengthening assessment processes to ensure the competence of newly qualified nurses.

The literature recognised the power imbalance between students–assessor relationships and the complexities in delivering student feedback (Rees et al. [28]; Borren et al. [24]; Natterøy et al. [15]). Assessors were conscious of the power they exerted over students (Borren et al. [24]; Natterøy et al. [15]), but they also encountered students exerting their own power by opposing assessor authority or responding with challenging, manipulative or abusive behaviour, consistent with previous studies (Burgess et al. [64]; Duffy [55], Hughes et al. [13]; Rees et al. [28]). Dweck and Yeager [65] (pp. 484‐485) explore ‘mindset theory’ to explain variations in student responses to feedback; however, the feedback is delivered constructively. Learners who possess a good development attitude, emotional stability, responsibility and sociability are more motivated to actively seek and use feedback constructively. However, learners frequently underestimate their capacity to assume accountability for their own learning (King [66]). Negative assessments may result in shock, disbelief, rage, denial and blame from students, influenced by lack of insight, inaccurate self‐evaluation and past assessors incorrectly passing prior placements (King [66]; Duffy [39]; Hughes et al. [13]; Rees et al. [28]).

The literature extensively documents the emotional toll that assessors experience when they fail a student, with participants describing it as stressful, horrendous, traumatic and draining (Watson and Harris [38]; Duffy [39]; Duffy [55]; Hunt [67]; Rodger and Juckes [29]; Mathisen et al. [27]; Natterøy et al. [15]). Assessors rely heavily on their own emotional resilience to manage conflicting feelings (Hunt [67]). Assessors highlighted the need to mentally prepare oneself to ‘grasp that nettle’ when failing a student, identifying the need to be courageous, bold, assertive and undaunted in the face of opposition, noting that speaking up can feel dangerous or futile (Scholes and Albarran [16]; Hunt [67]).

Barriers to giving effective feedback included the fear of upsetting the student, student resistance, defensiveness and a lack of respect for assessors (Hesketh and Laidlaw [68]). Adkins and Aucoin [14] examined face‐implicating factors, or the ‘Mum effect’, finding themes consistent with previous research on this topic (Dibble [36]; Dibble and Sharkey [37]). The MUM effect, known as ‘keeping mum’, describes the difficulty and hesitation of delivering unwelcome news. The ‘MUM effect’ is motivated by the messenger’s fear of being viewed in a negative way, being blamed and causing distress to the recipient, which can impede the efficient communication of bad news (Dibble [69]). Adkins and Aucoin [14] concluded that participants who had not committed failure to fail were less affected by face‐implicating factors. Hunt [67] identified the essential characteristics that robust and effective assessors need to possess, which include solidarity, tenacity, audacity, dependability and integrity. Assessors must confidently engage in difficult conversations and fail students when necessary (Duffy [55]). While selecting assessors with these specific characteristics may enhance assessment processes and reduce failure to fail (Hunt [67]), this could restrict the assessor pool, potentially impeding the students’ placement experience.

### 4.2. Strengths and Limitations

Consultation with an information specialist enhanced the search’s replicability and breadth, by clarifying key terms and suggesting suitable databases (Cooper et al. [21]). Despite this assistance, it is feasible that pertinent papers may not have been found. The terminology to describe student nurses and assessors differed geographically, and despite including several variations in the search, a relevant term may have been omitted. This review was undertaken by one researcher with supervisor guidance. The author acknowledges that a second reviewer would have improved accuracy, efficiency and reduced the risk of bias (Stoll et al. [70]).

## 5. Conclusions and Recommendations

Failure to fail remains a well‐researched and internationally recognised concept in nursing and healthcare despite variations in assessment methods. In the UK, NMC reforms (NMC [4]; NMC [2]) transformed the methods of mentorship in nursing education. Despite the scarcity of national research to assess the effectiveness of these standards and the impact on failure to fail, findings from Whaley, Hay and Knight [30] align with existing international literature.

This review has identified the impact of inadequate assessor education, time constraints, motives for reluctance to provide feedback, and relationship and power dynamics.

The nursing profession must acknowledge the serious consequences of allowing incompetent students to graduate. Assessors who fail to fail neglect their duty of care to patients, students and the profession itself. Implementing recommendations from this review could enable regulatory bodies, educational institutions and placement providers to significantly reduce the barriers and better support assessors in making difficult, yet necessary decisions. Equipping assessors with the necessary knowledge, support and resources to be effective gatekeepers to the nursing profession is crucial for cultivating a high‐quality, future workforce. However, delivering high‐quality care also requires significant investment—a timeless principle Florence Nightingale affirmed:

‘For the sick, it is important to have the best’ (Great Britain Parliament House of Commons [71], p. 343).

### 5.1. Implications for Research

The following recommendations are derived from the findings of this review and are supported, where appropriate, by relevant policy and published literature.

• This review identified a lack of recent UK‐based research examining assessors’ experiences of failing underperforming student nurses. There is a need for more comprehensive national data to draw robust conclusions about the assessor experience in the UK. Enhanced data collection could inform targeted support strategies for educators, ultimately improving student supervision and learning environments (NHS England [72]).

### 5.2. Implications for Education

• Findings from this review highlighted persistent gaps in assessors’ understanding of assessment processes, documentation requirements and the interpretation of competence. These findings support the need for robust, standardised assessor preparation and regular updating opportunities. Assessors should be enabled to access ongoing education, including training in performance management, formative assessment and the delivery of constructive feedback, to enhance assessor confidence and assessment quality.

### 5.3. Implications for Policy and Practice

• Consistent with findings that workload pressures and time constraints negatively impacted assessment practices, allocating protected time for assessors could alleviate workload pressures, reduce stress and burnout, and support the delivery of accurate, timely and defensible assessments. This may also help minimise student appeals and address the issue of failure to fail (NHS England [62]).
• Additional support is needed for assessors, particularly when managing underperforming students. Strengthening and expanding the role of practice educators could offer a cost‐effective solution by improving assessment processes, reducing supernumerary time and preventing the long‐term costs of poor practice.

## Funding

This review did not receive any specific grant from funding agencies in the public, commercial or not‐for‐profit sectors.

## Conflicts of Interest

The authors declare no conflicts of interest.

## Data Availability

The data that support the findings of this study are available from the corresponding author upon reasonable request.
